# Sustainable valorization of waste polystyrene and PVC blends for high-performance applications

**DOI:** 10.1038/s41598-026-49599-5

**Published:** 2026-05-05

**Authors:** Emad S. Shafik

**Affiliations:** Polymers and Pigments Department, Chemical Industrial Research Institute, National Research Centre, Dokki, P.O.12622, Giza, Egypt

**Keywords:** Waste polystyrene foam, Polyethylene terephthalate, Glycolysis, Mechanical properties, Dielectric properties, Chemistry, Energy science and technology, Engineering, Materials science

## Abstract

Disposal of solid plastic waste is one of the major challenges facing most countries, especially developing countries. In this study, waste polystyrene foam (WPS) and polyethylene terephthalate (PET) from waste plastic bottles were recycled. The waste PET was chemically recycled using a glycolysis technique to obtain the glycolyzed product (GPET) and subsequently unsaturated polyester. WPS was also mechanically recycled using a hot blending technique. Polyvinyl chloride, GPET, and the prepared polyester were added as multifunctional additives and their effects were compared with conventional additives such as dioctyl phthalate (DOP). The effect of these additives on the rheological, mechanical, and electrical properties was studied. Thermal stability, fire retardancy and morphology of the WPS/PVC blends were also evaluated. Results showed that polyester significantly improved melt flow rate (up to 0.975 g/10 min), flame retardancy (burning rate reduced to ~ 0.5 mm/s), and decreased combustion energy (~ 404 cal). GPET enhanced tensile strength (21.96 MPa) and elongation at break (37%). Additives increased permittivity and conductivity, while polyester exhibited the strongest dielectric response due to enhanced interfacial polarization. SEM analysis confirmed better dispersion and compatibility in GPET and polyester blends. Based on the results, the developed blend shows strong potential for use in applications requiring enhanced mechanical, thermal stability, and flame retardancy as electrical insulation, construction materials, and automotive components.

## Introduction

The global proliferation of plastic materials across diverse industrial sectors is largely attributable to their cost-effectiveness, low density, corrosion resistance, and ease of fabrication^1–3^. These characteristics have rendered synthetic polymers indispensable in applications ranging from packaging and automotive components to construction, electronics, and biomedical devices^4–7^. However, the escalating consumption of plastics has resulted in a parallel increase in polymeric waste, which presents a critical environmental concern due to the recalcitrant nature of most conventional plastics to biodegradation^8–12^.

Among the predominant thermoplastics in commercial use are polyethylene (PE), polypropylene (PP), polyvinyl chloride (PVC), polyethylene terephthalate (PET), and polystyrene (PS)^13–15^. These materials are valued for their unique mechanical and chemical properties, including flexibility, durability, and resistance to moisture and solvents. Nevertheless, their persistence in the environment has prompted substantial research into sustainable end-of-life solutions, including mechanical recycling, chemical depolymerization, and the development of biodegradable alternatives^16^.

Expanded polystyrene (EPS), a rigid, lightweight, and closed-cell foam derived from polystyrene, is extensively utilized due to its superior thermal insulation, impact resistance, and hydrophobicity^17–19^. It is commonly employed in protective packaging, food containers, and building insulation. Despite these advantageous properties, EPS poses significant recycling challenges owing to its high volume-to-mass ratio and low economic return in post-consumer recycling streams^20^. Recycling methods such as mechanical densification and chemical depolymerization have been explored to recover EPS for secondary applications, including the production of insulation boards, molded products, and decorative materials^21,22^.

Polyethylene terephthalate (PET), particularly in the form of beverage bottles, represents another major source of post-consumer polymeric waste. Chemical recycling via glycolysis—a process wherein PET is depolymerized using glycols such as ethylene glycol yields glycolyzed PET (GPET), a valuable intermediate for the synthesis of unsaturated polyesters or as a functional modifier in polymer blends^23–25^. The incorporation of GPET in polymer matrices has been shown to enhance thermal stability, mechanical performance, and environmental compatibility of the resulting composites^16^.

Polymer blending is an effective strategy to improve the performance and sustainability of composites by synergistically combining the properties of distinct polymers^26^. The current research aims to eliminate non-biodegradable plastic wastes that negatively impact the environment, such as polystyrene (PS) and polyethylene terephthalate (PET), by recycling each of them. The former was mechanically recycled by blending it with polyvinyl chloride (PVC) to improve rigidity, thermal resistance, and processability, while the latter was chemically recycled through glycolysis process, and the glycolyzed product was used to produce unsaturated polyester. The glycolyzed product and prepared polyester were also used as additives to the WPS/PVC blend, and the results were compared with DOP to improve their mechanical and electrical properties to be suitable for various industrial applications.

## Material and experimental techniques

### Materials and raw materials

Ethylene glycol M.Wt 62.07 g/mol and Adipic acid M.Wt 146.14 g/mol were purchased from Alpha Chemika, India. Zinc acetate M.Wt 183.48 g/mol - d. 1.84 g/cm^3^, phthalic anhydride M.Wt 148.12 g/mol- d. 1.53 g/cm^3^, tin(II) ethylhexanoate M.Wt 405.12 g/mol- d. 1.251 g/cm^3^ were obtained from Aldrich. Polystyrene waste was collected from Egyptian landfills, washed with distilled water and left to dry. Silica filler was obtained from Transport and Engineering Company, Alexandria, Egypt. Inovyn Polyvinyl chloride resin powder, K-Value 60, Made in Spain was utilized in the study.

###  Glycolysis of waste PET

The PET was obtained from waste soft drink bottles, which were collected, washed and cut into small pieces. It was then washed and dried. PET and ethylene glycol (1:4, w/w) were charged into the reactor in the presence of 0.5%zinc acetate catalyst.

###  Preparation of polyester from GPET

**Scheme 1:**
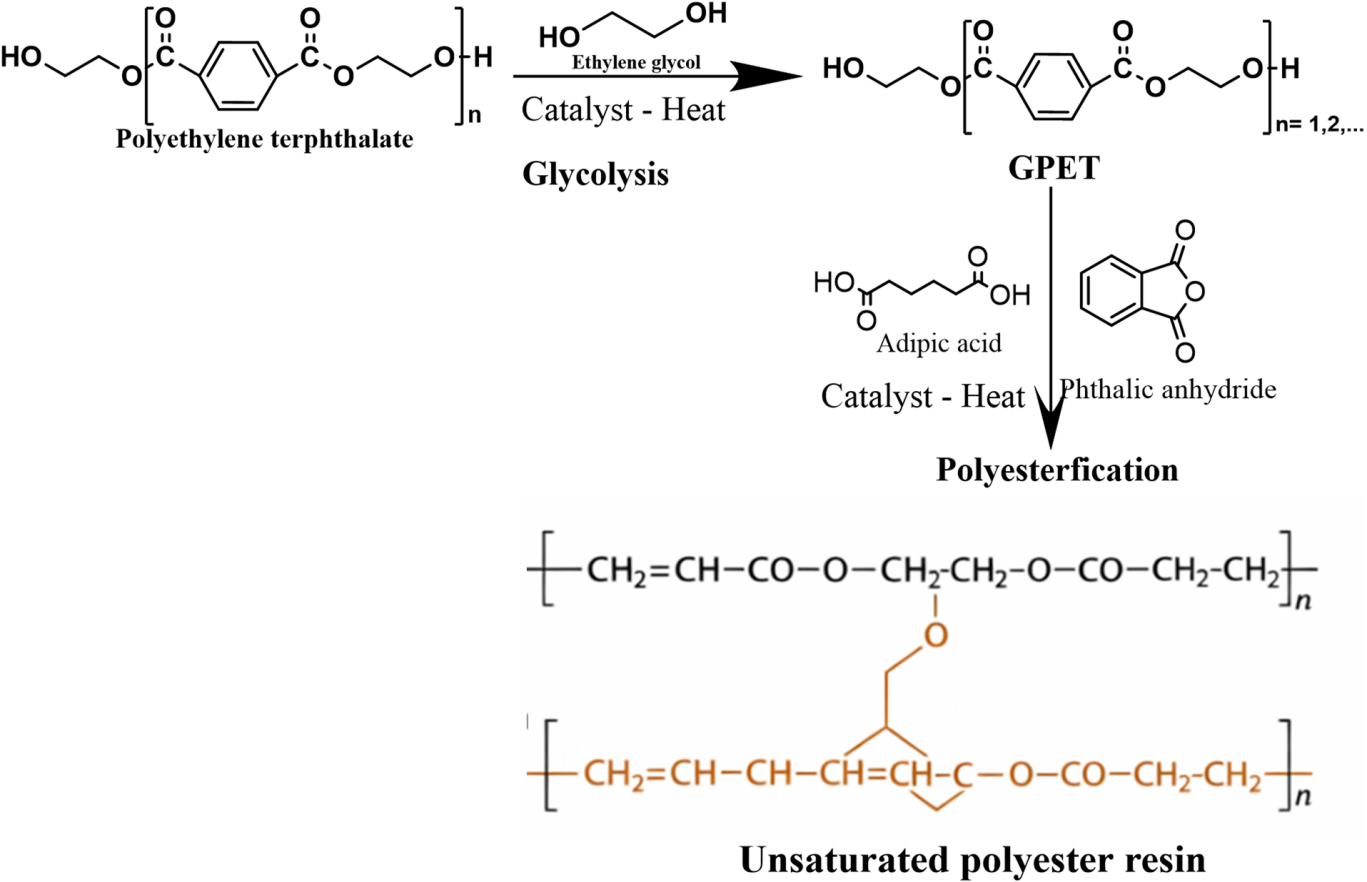
Glycolysis of PET and subsequent synthesis of unsaturated polyester resin.

Unsaturated polyester based on GPET and adipic acid was synthesized through esterification reaction as shown in Scheme 1. The esterification reaction was carried out in a reactor equipped with a stirrer, thermometer, nitrogen gas inlet, and reflux set at 180–190 °C. Adipic acid (21.2%) and phthalic anhydride (3.1%) were added to GPET as reactant (75.2%) in the presence of 0.5% of tin (II) 2-ethylhexanoate as a catalyst. Both GPET and prepared polyester were characterized in our previous research^14^.

### Blending waste polystyrene/polyvinylchloride blends

Waste polystyrene, and polyvinyl chloride were mixed with constant weight ratio (80:20) in an internal mixer (Brabender Plastic order) at 30 rpm and temperature of 170 °C for waste polystyrene formulations. First, waste polystyrene was added to the mixer then polyvinyl chloride and silica filler 10% was added after the polymers had reached the melting temperature. DOP, GPET and prepared polyester with fixed content 2.5% were added to PS/PVC blends to determine their efficiency on mechanical and dielectric properties. The mixing process took 5 min on average. After the resultant blends were removed from the mixer, pressed into different dimensions using laboratory hydraulic hot press at 170 °C for 5 min, and then cooled down to room temperature.

###  Characterization of waste polystyrene/polyvinylchloride blends

####  Rheology and mechanical properties

The melt flow indices (MFI) of PS/PVC blends were determined as per ASTM D 1238 at 190 °C with a 2.16 kg load, using a Zwick MFLOW instrument. Tensile strength and elongation at break for the prepared composites were evaluated through Zwick tensile testing machine (model Z010, Germany), according to ASTM D638 standard. In addition to hardness (shore D) was evaluated according to ASTM D2240, using Hardness tester Model HR-150 A Rockwell. The test is based on the penetration of the durometer indentor when forced into the material. Subsequent readings at different points were taken after the indenter made contact with the specimen.

For each MFI measurement, the reported value represents the average of three specimens, whereas all mechanical properties (tensile strength, elongation at break, and hardness Shore D) were calculated as the mean of five independent specimens to ensure statistical reliability.

####  Thermal and structural stability

Thermogravimetric analysis (TGA) was performed using TA instrument 2910 series. The heating rate was 10 °C/minute in nitrogen environment from room temperature up to 600 °C. Differential scanning calorimetric (DSC) was carried out under oxygen flow using a Q20 differential scanning calorimeter, USA. The operating temperature ranges from ambient temperature up to 500 °C.

####  Fire retardancy test

The burning rate was measured according to ASTM D 635-03 for three specimens. The dimension of the specimen was (100 mm L* 13 mm W* 6 mm Thick). The time of the drip was recorded.

####  Phase morphology

Phase morphology was observed by using scanning electron microscope (SEM) Quanta instrument (model FEG250, FEI, Hillsboro, OR, USA). The samples were manually cut, fixed on stubs with double-sided tape and coated with gold.

####  Dielectric measurements

Dielectric measurements, the samples were prepared with diameter of 5 cm and a thickness of 1.2 mm. The capacitance C, loss tangent tan δ and the resistance R were determined in the low frequency range (100 Hz to 100 kHz) using an LCR meter type AG-411 B (Ando electric Ltd., Japan). The higher frequency ones (100 kHz to 120 MHz) the used apparatus was an LCR HiTESTER 3535 Model. MC-100 cell was used for both instruments. The permittivity ε′ was estimated from the well-known relation ε′ = C/Co where C is the sample capacity and Co is that in of air. The dielectric loss ε″ was obtained from the multiplication of the ε′ values by the loss tangent tanδ^27^.

## Results and discussion

### Effect of additives on rheological and mechanical properties of WPS/PVC blends

**Fig. 1:**
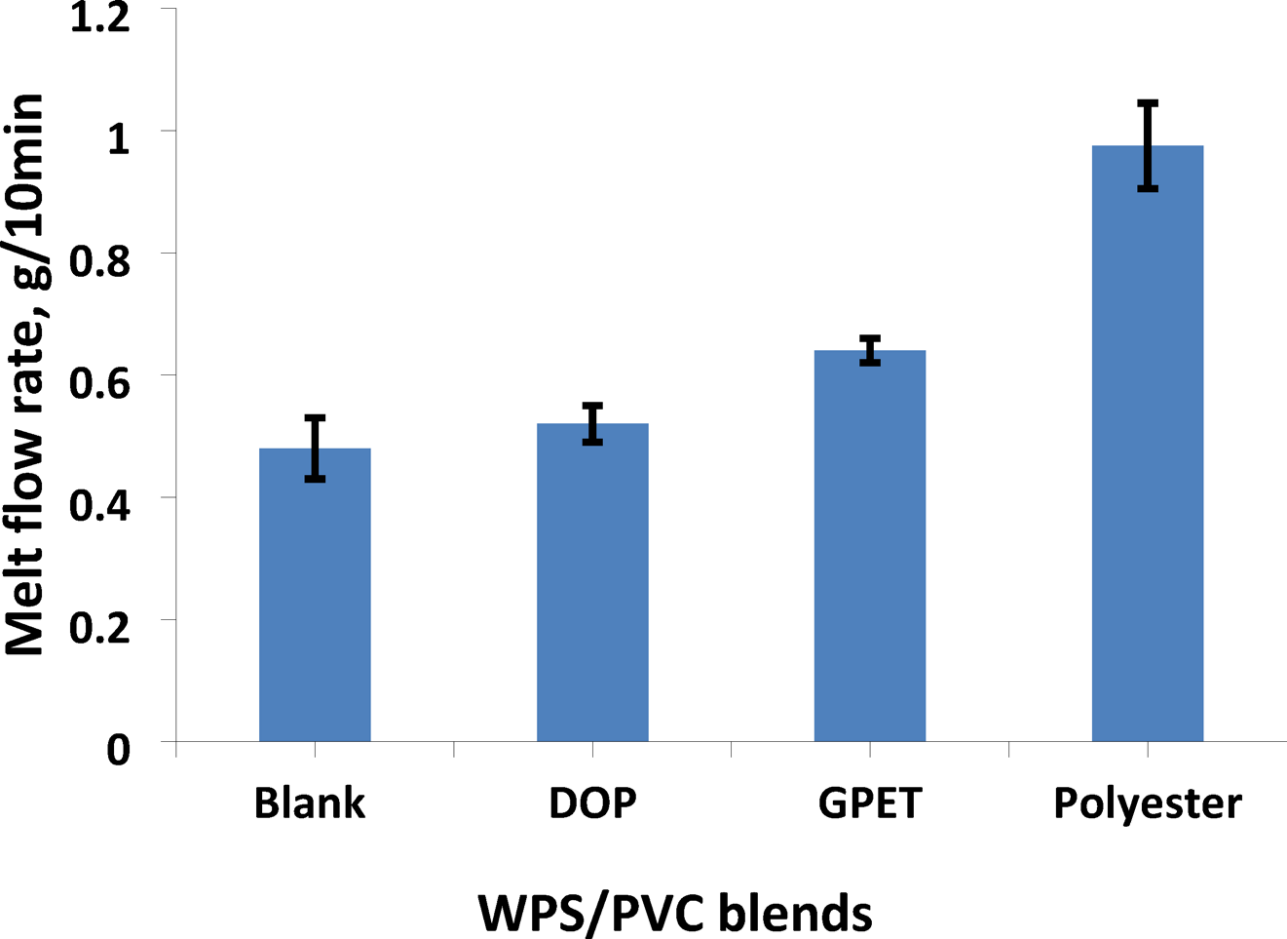
Melt flow rate of WPS/PVC blends with additives.

Figure 1 represents the variation in melt flow rate of WPS/PVC blends incorporating different additives, including DOP, GPET, and prepared polyester. The figure illustrates that the additives increase the melt flow rate of the blends. The melt flow rate increased from 0.48 g/10min for the blank sample to 0.52, 0.64 and 0.975 g/10min for WPS/PVC containing DOP, GPET, and polyester respectively. The slight increase in melt flow rate obtained with DOP aligns with earlier findings showing that low molecular weight plasticizers reduce intermolecular interactions in PVC but produce only modest improvements in flow at typical loadings^28,29^. In contrast, the more pronounced increase observed with GPET agrees with reports indicating that glycolyzed PET oligomers act not only as polymeric plasticizers but also as compatibilizers that enhance interfacial adhesion and promote a more homogeneous melt morphology^30^. The highest melt flow rate recorded for the polyester-modified blend is also in good agreement with previous studies showing that polyester-based compatibilizers significantly improve processability by reducing interfacial tension and lowering blend viscosity^31^. Overall, the results confirm that compatibilization and improved phase interaction have a stronger influence on melt flow enhancement than conventional plasticization alone.

**Fig. 2:**
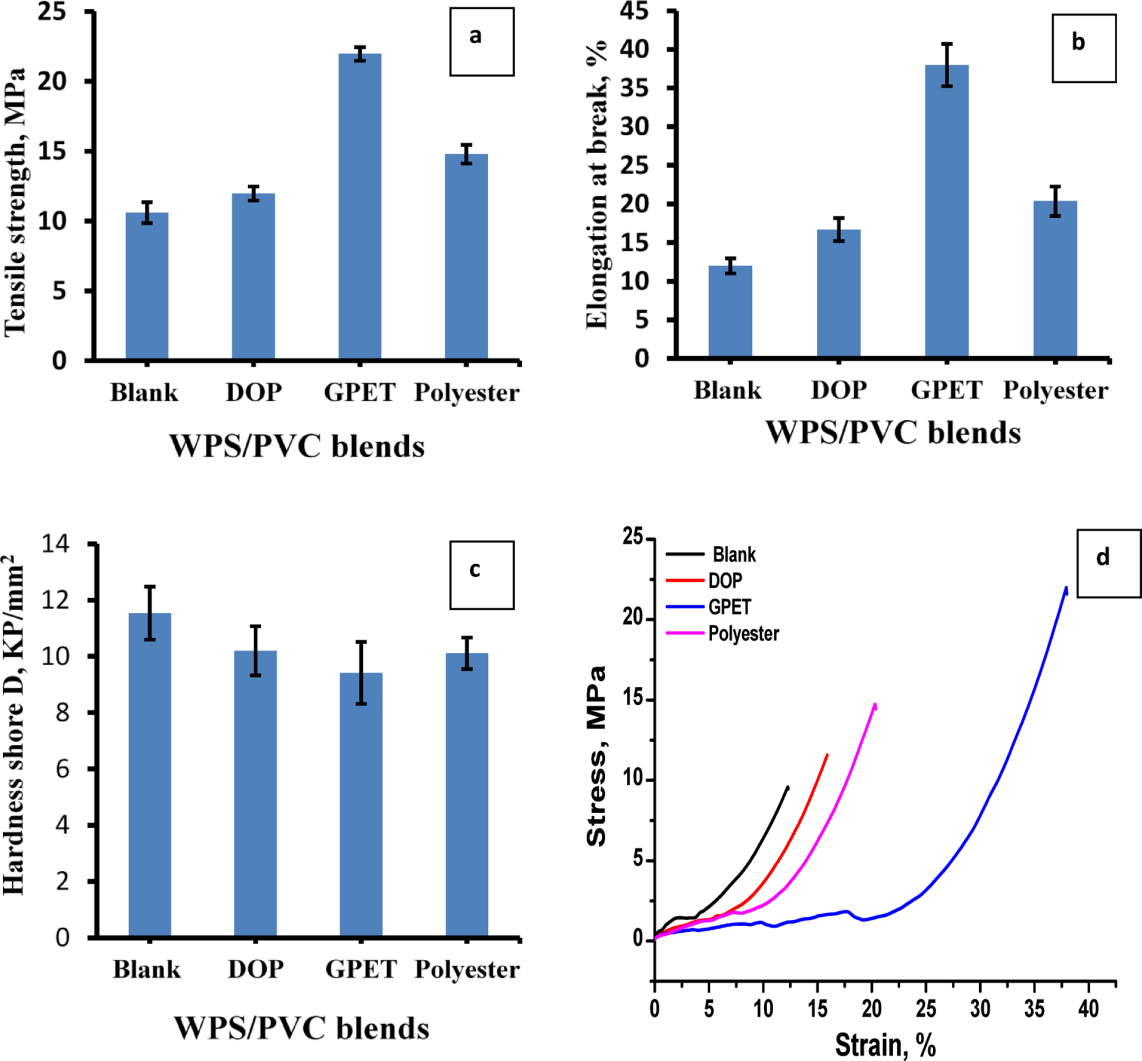
Mechanical properties of WPS/PVC blends.

Figure 2(a-d) illustrates the mechanical properties of PS/PVC filled with 10% silica containing different additives such as DOP, GPET and prepared polyester. Figure 2a represents the relation between tensile strength MPa versus different additives. It is clear that these additives increase tensile strength of the polymeric blends where the tensile increased from10.6 MPa for blank sample to be 11.97, 14,78 and 21.96 MPa for PS/PVC containing DOP, Polyester, and GPET. The slight increase in tensile strength observed with DOP agrees with traditional behavior of low-molecular-weight phthalates plasticizers. DOP increases the chain mobility of polymer chains and weakens cohesive intermolecular forces, leading primarily to a plasticization effect. Because DOP has a relatively low molecular weight and limited polarity, its interaction with the PS/PVC blend is mainly physical, resulting in enhanced flexibility and limited tensile improvement^32,33^.

Unlike DOP, GPET and the prepared polyester possess higher molecular weight and contain multiple polar ester functional groups capable of forming dipole–dipole interactions with PVC chains and possible π–π interactions with the aromatic rings of PS. These interactions enhance both intermolecular cohesion and stress transfer across between phases. Furthermore, their semi-polymeric nature allows them to act not only as internal plasticizers but also as compatibilizers, reducing interfacial tension between WPS and PVC phases and improving dispersion of silica particles. Similar observations have been reported for PET-derived plasticizers, where glycolyzed PET enhances tensile properties due to strong polar interactions and improved phase compatibility in PVC systems^34^. Similarly, Amaro et al. reported that PET-derived polyester secondary plasticizers improve mechanical stability of polymer blends by acting as compatibilizers that enhance interfacial adhesion^35^.

Figure 2b also shows the relationship between the elongation at break and the different additives. From this figure, it is clear that the elongation values of the WPS/PVC blends increased with the addition of different additives compared to the blank sample. Elongation at break increased from 12% for the blank to become 17, 20 and 37 for the blends containing DOP, polyester and GPET, respectively. The main reason for the increase in elongation when using polyester and GPET is due to their ability to increase flexibility and mobility of the polymer chains more than DOP.

As shown from Fig. 2c, hardness shore D values decrease with the addition of additives to PS/PVC blends. The blank sample shows the highest hardness which was 11.54 KP/mm^2^, while the addition of plasticizers slightly reduces the hardness to be 10.2, 9.41, 10.11 for DOP, GPET and polyester. This decrease is due to the effect of plasticizers, which reduce hardness by disrupting the packing of polymer chains^32^. However, the moderate reduction observed for GPET and polyester compared to DOP suggests that these additives partially compensate for softening through enhanced intermolecular interactions and improved phase cohesion.

**Fig. 3:**
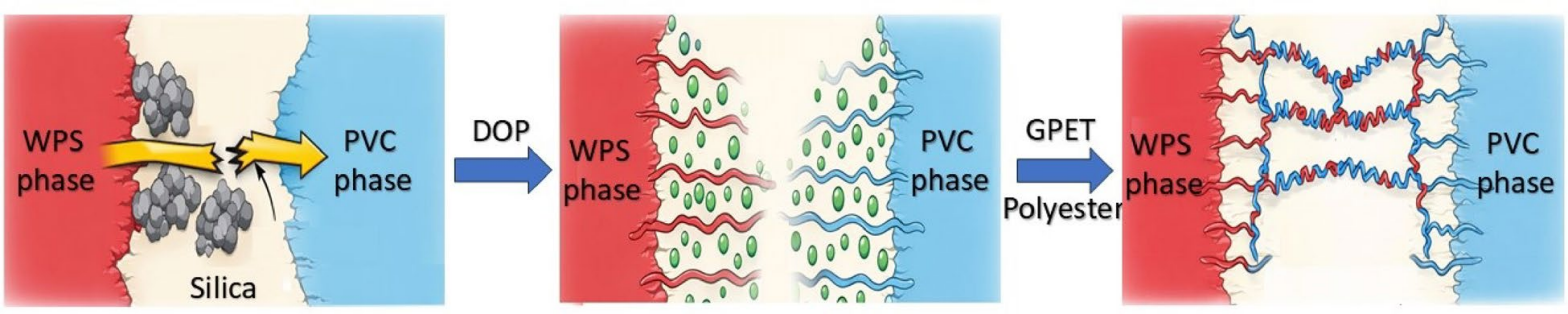
Proposed dual plasticizing and compatibilizing mechanism of additives in WPS/PVC blends. The schematic illustration was manually created by the authors using Microsoft PowerPoint based on the experimental observations and interpretation obtained in this study. No AI-based tools were used.

Also, Fig. 2d illustrates stress-strain curve for WPS/PVC composites. From this figure, it is clear that the blank sample has less stress strain and this is due to absence of plasticizer. While WPS/PVC composites plasticized with GPET and polyeseter exhibit superior strain tolerance and stress-handling capabilities, indicating improved toughness. The performance of DOP falls between the blank and GPET/Polyester, emphasizing its moderate enhancement of properties. The proposed mechanism of additives in WPS/PVC blends was illustrated in Fig. 3.

### Effect of additives on thermal stability and fire retardancey of WPS/PVC blends

The oxidation induction time (OIT) is a standardized assessment performed via differential scanning calorimetry (DSC). This test evaluates the thermal stabilization of the material by measuring the interval between melt flow and the onset of decomposition under isothermal circumstances^27^.

The Arrhenius equation delineates the relationship between energy and reaction rate as follows:

1 $$(\Delta {\mathrm{A}}/\Delta {\mathrm{t}})\,=\,{\mathrm{k}}{{\mathrm{e}}^{ - {\mathrm{E}}/{\mathrm{RT}}}}$$

Where:

t= Reaction time k= Reaction constant.

E= Energy of the reaction R= Universal gas constant.

T= represents absolute temperature in Kelvin (K).

From Eq. (1), the subsequent equation is derived:

2 $${\text{Ln }}(\Delta {\mathrm{A}}/\Delta {\mathrm{t}})\,=\,{\mathrm{lnk}} - {\mathrm{E}}/{\mathrm{RT}}$$

Equation (2) can be adjusted as follows:

3 $${\mathrm{ln}}f{\mathrm{t}}\left( {{\mathrm{OIT}}} \right)={\text{ const}} - {\text{ E/RT}}$$

**Fig. 4:**
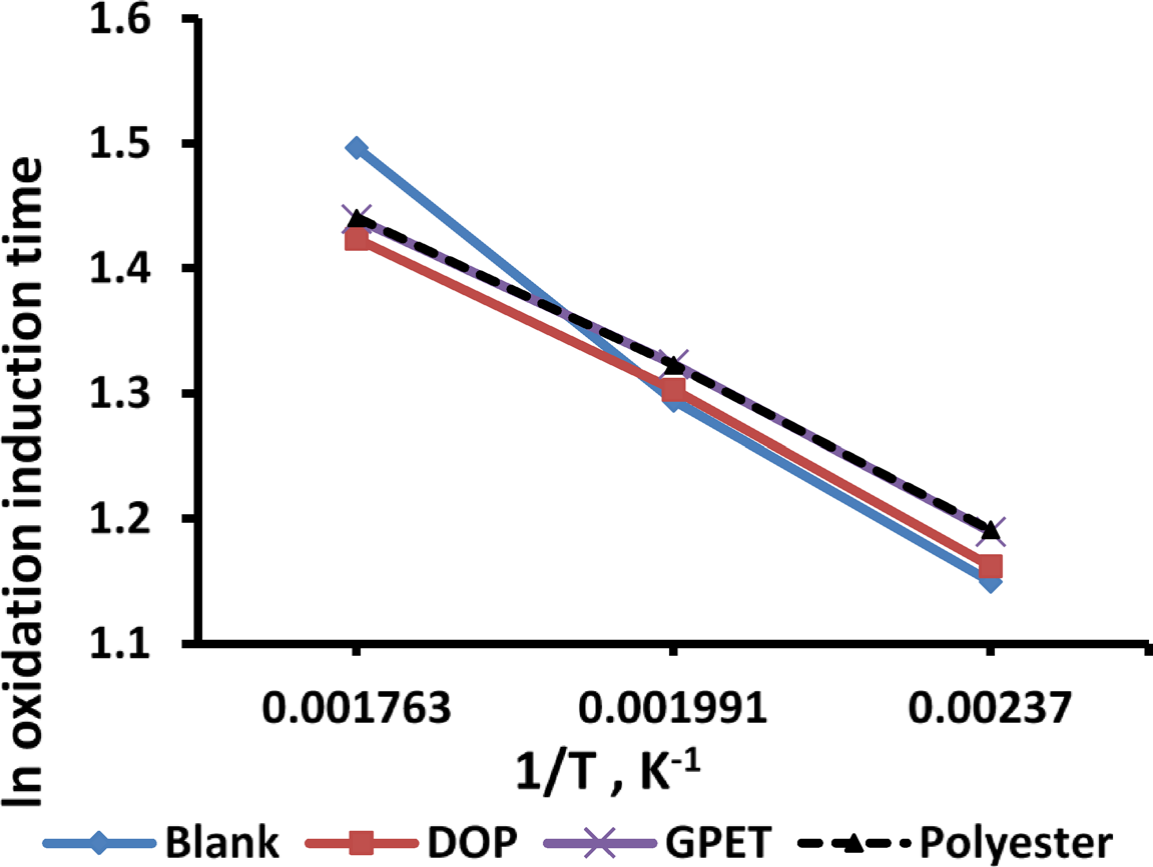
Relation between ln oxidation induction time and the reciprocal value of absolute temperature for WPS/PVC blends.

Figure 4 illustrates the straight line relationship between logarithm oxidation induction time (ln OIT) as a function of the reciprocal temperature (1/T) for various WPS/PVC blends containing additives (DOP, GPET, and Polyester) Also, Table 1 illustrates the numerical data of ln OIT at different temperature. The OIT values decline with rising temperature, signifying a reduction in thermal stability. Among the composites, the blank sample (without any additives) demonstrates the lowest oxidation stability, whereas the blends containing GPET and polyester show markedly higher OIT values. This enhancement aligns with previous studies demonstrating that PET-derived oligomers and polyester additives can improve oxidation resistance in polymer blends by promoting char formation and stabilizing the polymer matrix during heating^36,37^. Their aromatic structure and ester groups are known to inhibit radical chain reactions, thereby prolonging the induction time before oxidative degradation starts.

**Table 1:** ln oxidation induction time for WPS/PVC blends.

Tem. (ᵒC)	1/T (K^− 1^)	ln oxidation induction time for WPS/PVC blends			
		Polyester	GPET	DOP	Blank
294.2	0.001763	1.496653	1.423737	1.440594	1.439806
229.2	0.001991	1.294466	1.303196	1.322839	1.323665
174	0.00237	1.149835	1.161967	1.191171	1.189771

**Fig. 5:**
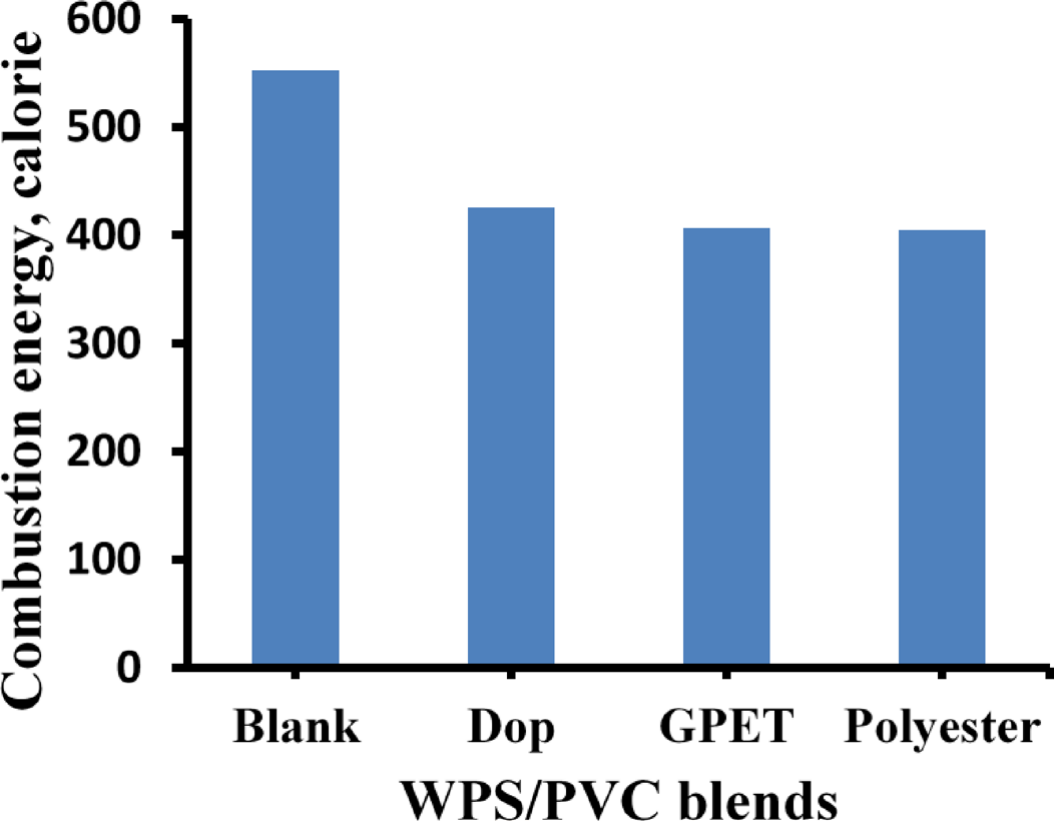
Combustion energy for WPS/PVC blends.

Combustion energy (in calories) for different WPS/PVC can be calculated from the slope of the relation between ln OIT and 1/T and plotted as in Fig. 5. The blank composite has the highest combustion energy (~ 552 cal), followed by samples with DOP, GPET, and polyester, which were 425.3, 405.96, and 404.46, respectively. This suggests that the additives effectively lower the energy required for combustion, contributing to fire retardancy. The reduction in combustion energy implies that these additives suppress flammability by interfering with the combustion process^27,38^.

**Fig. 6:**
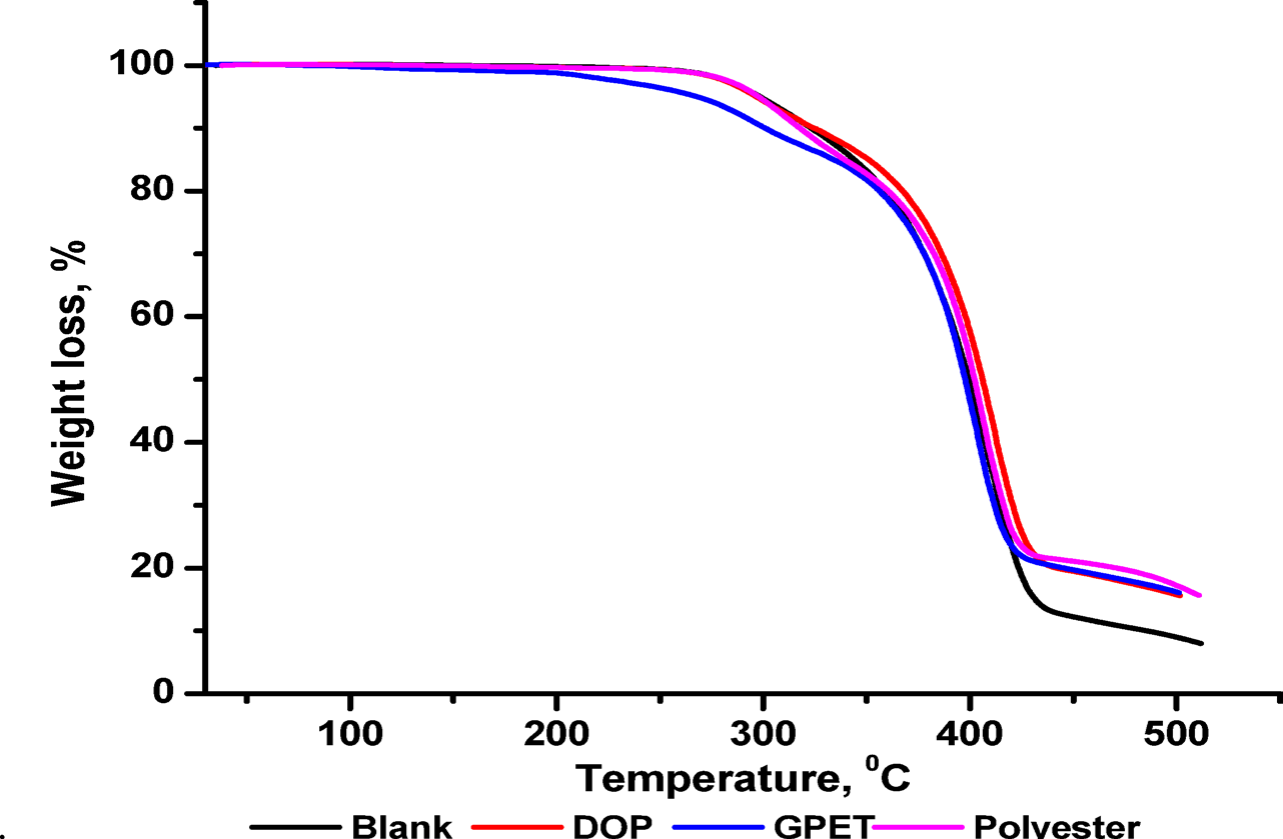
Thermo gravimetric analysis of WPS/PVC blends.

Figure 6 demonstrates the thermal gravimetric analysis of WPS/PVC blends with additives. Also, Table 2 lists the thermal degradation of blends. It is clear that, the blank sample without any additive, demonstrates the lowest thermal stability with T₁₀% recorded at 325.75 °C and the lowest final residue (8.8%).Adding DOP results in a slight increase in T₁₀% (326.67 °C) and a substantially higher residue yield (16.01%), indicating a moderate improvement in thermal stability due to its plasticizing effect, which facilitates improved heat transfer but its stabilizing contribution is limited. On the other hand, GPET and prepared polyester exhibit better thermal stability compared to DOP. WPS/PVC plasticized with GPET show an earlier onset of weight loss (T₁₀% = 298.48 °C), but a higher T₂₀% (354.87 °C) and increased residue (16.54%), suggesting improved structural integrity during the major degradation stage. Furthermore, synthesized polyester demonstrates the most significant enhancement among all additives, confirmed by higher T₁₀% (317.27 °C), elevated T₂₀% (359.57 °C), and the highest residue yield (17.06%).WPS/PVC plasticized with GPET and polyester show a more gradual degradation behavior and higher char residue formation compared to both the blank and DOP-plasticized samples. This result confirms their superior thermal stability and their efficiency as fire retardant^39^.

**Table 2:** Thermal degradation of WPS/PVC blends.

Plasticizers	Tem. (℃)			Residue wt% at 500℃
	T10%	T20%	T50%	
Blank	325.75	357.7	399	8.8
DOP	326.67	366.11	406	16.01
GPET	298.48	354.87	397.1	16.54
Polyester	317.27	359.57	401	17.06

**Fig. 7:**
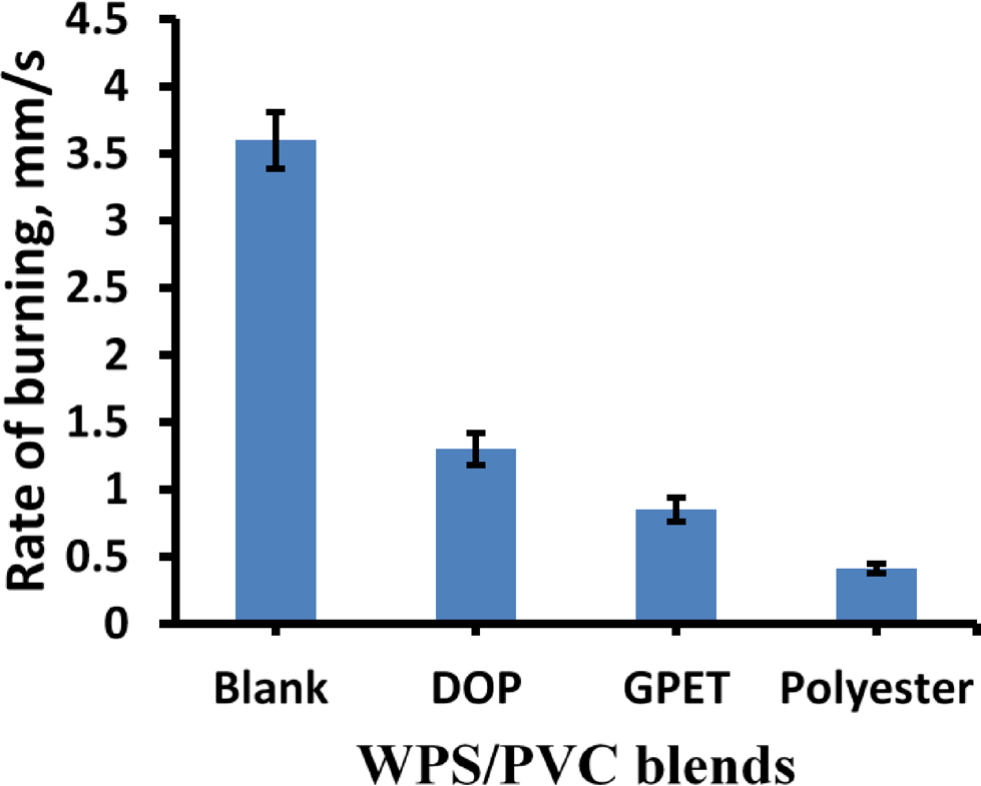
Rate of burning mm/s for WPS/PVC blends.

Figure 7 shows the rate of burning (mm/s) for WPS/PVC composites. The blank sample exhibits the highest burning rate (~ 4 mm/s), while the addition of DOP, GPET, and Polyester significantly reduces this rate, with Polyester showing the most notable suppression (~ 0.5 mm/s). This confirms the flame-retardant nature of GPET and Polyester.

### Effect of additives on surface and cross section of WPS/PVC blends

**Fig. 8:**
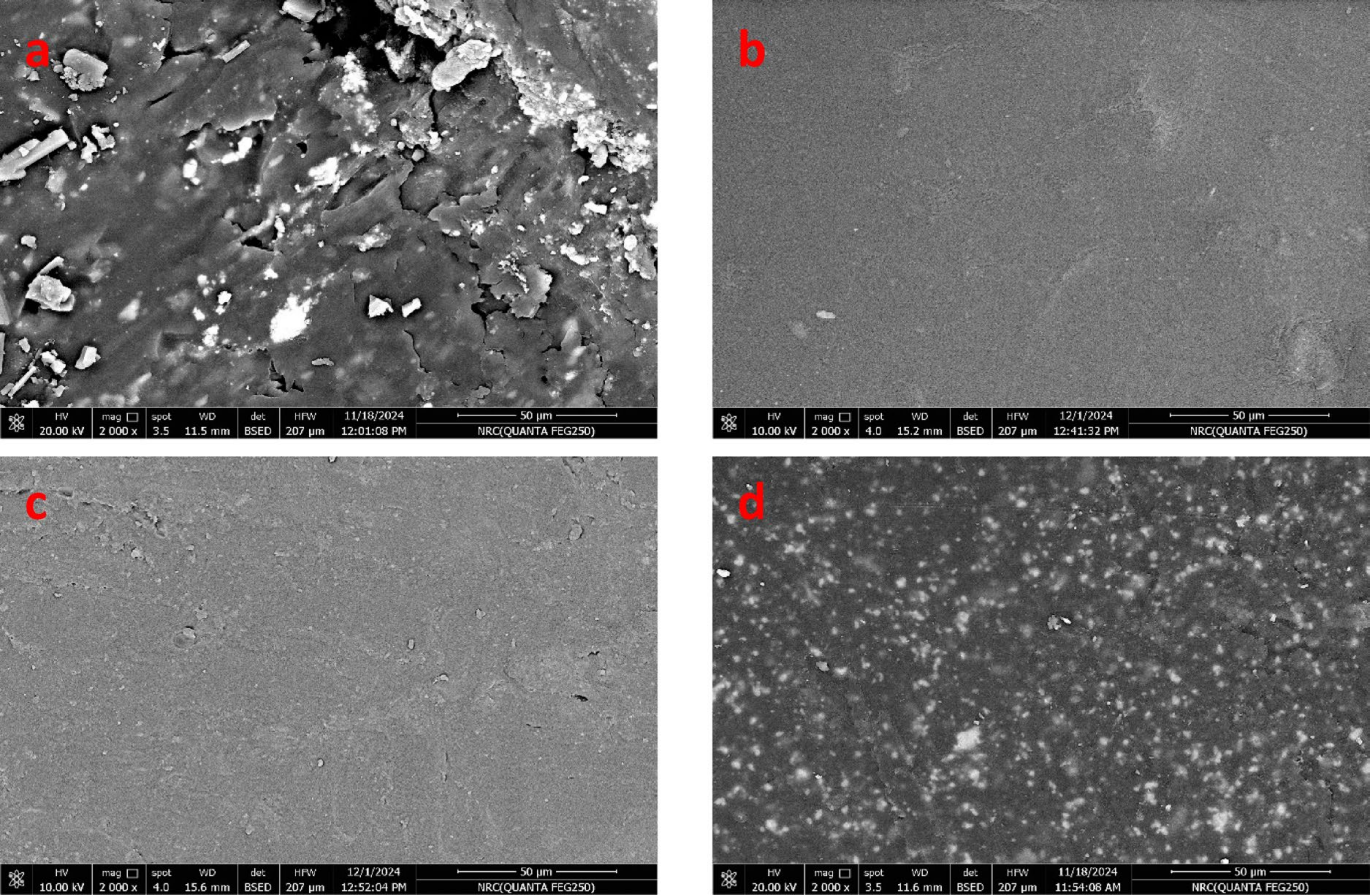
Scan electron microscope of surface WPS/PVC blends. (**a**) blank, (**b**) Dop, (**c**) GPET, (**d**) Polyester.

Figure 8 (a-d) illustrates the surface morphology of WPS/PVC blends. From Fig. 8a, it is clear that the surface of WPS/PVC filled with silica without additives appears irregular and fragmented, with a dispersed distribution of silica particles. This suggests poor dispersion and adhesion between silica and the polymer matrix.While after adding DOP to the mixture Fig. 8b, it was observed that the surface became more uniform, suggesting that DOP reduce phase separation and improve silica dispersion. Also, with the addition of GPET to the WPS/PVC blend as in Fig. 8c, the surface became relatively smoother, showed fewer cracks and a better integrated structure. Likewise the addition of GPET to the WPS/PVC blend as in Fig. 8d, surface became more uniform with fewer defects, indicating that polyester enhances polymer blend compatibility and provides better cohesion between phases^40^.

**Fig. 9:**
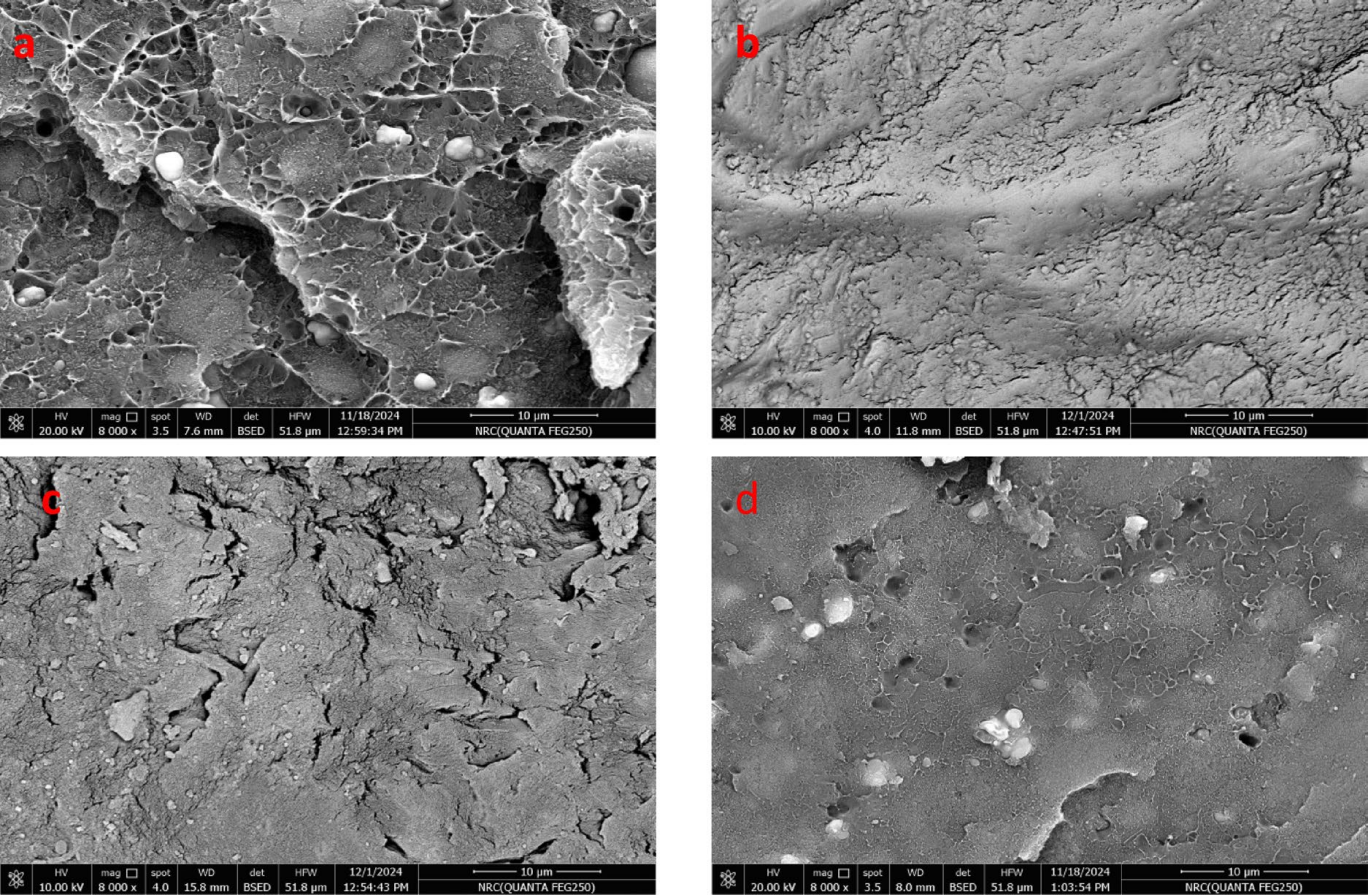
Scan electron microscope of cross section WPS/PVC blends. (**a**) blank, (**b**) Dop (**c**) GPET (**d**) Polyester

Also Fig. 9 illustrates the morphology of cross- section of WPS/PVC blends. From Fig. 9a, it can be seen that a rough and heterogeneous internal structure with visible phase separation. This confirms weak compatibility between WPS and PVC and poor dispersion of silica filler, leading to poor interfacial adhesion. After adding DOP the internal structure became smoother than blank sample as shown in Fig. 9b, indicating that DOP acts as a plasticizer and enhance the computability of blends. Furthermore, Fig. 9c illustrates the cross section structure of WPS/PVC blends plasticized with GPET. It is clear that the structure shows less porosity and improved homogeneity, indicating that GPET enhances compatibility between matrixes. Also Fig. 9d confirmed that the presence of polyester leads to a more continuous and less porous internal structure, showing improved adhesion between matrixes. SEM images clearly show improved compatibility and dispersion of the additives within the WPS/PVC blends, which directly corresponds to the observed increases in tensile strength and elongation at break. This demonstrates that the enhanced interfacial adhesion plays a vital role in enhancing the overall mechanical performance of the composites.

###  Effect of additives on dielectric properties of WPS/PVC blends

**Fig. 10:**
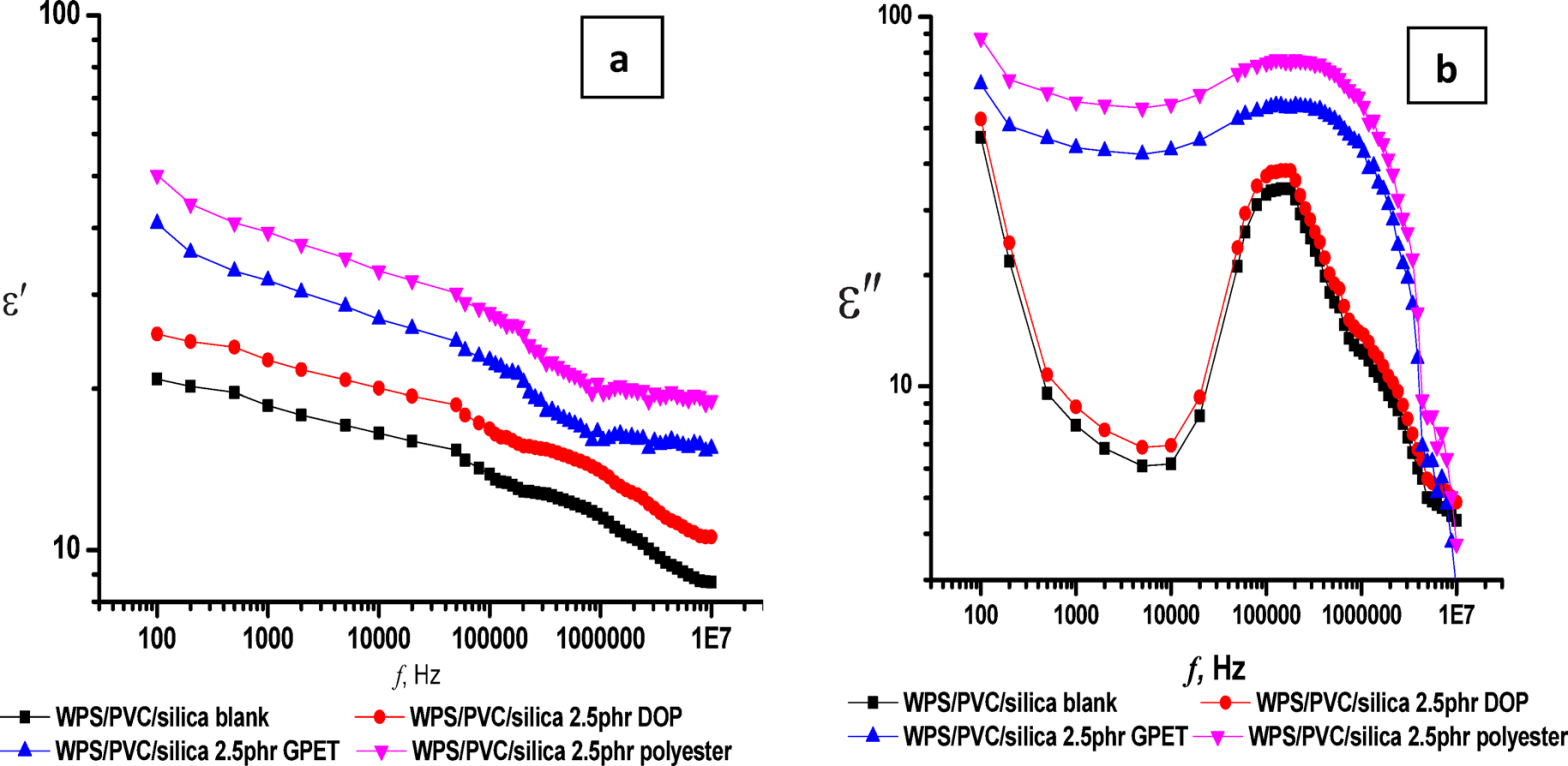
The permittivity ε′and dielectric loss ε″ for WPS/PVC blends.

The dielectric behavior of WPS/PVC blends filled with different additives DOP, GPET, and polyester was investigated across a broad frequency range (100 Hz up to 120 MHz) to assess the impact on permittivity and dielectric loss as shown in Fig. 10. As shown in Fig. 10a, the permittivity ε′ of all samples exhibited a decreasing trend with increasing frequency, consistent with the typical behavior of dipolar dielectric materials where polarization fails to follow the alternating field at high frequencies. The blank WPS/PVC blend without additives displayed the lowest ε′ values, while the addition of polar additives, particularly polyester, led to a notable increase in ε′ across the frequency spectrum. This enhancement is attributed to strengthened interfacial (Maxwell Wagners Sillars) polarization and increased dipolar contribution arising from the polar functional groups introduced by the additives.

The dielectric loss (ε″) revealed distinct relaxation peaks for all blends, with the blends containing polyester showing the most pronounced loss. This indicates greater energy dissipation and enhanced molecular mobility, likely due to stronger dipolar interactions and interfacial polarization mechanisms introduced by polyester. Typically, plasticizers can reduce the loss by improving molecular mobility and reducing intermolecular friction in WPS/PVC. In our case and as it its seen in Fig. 10, it is seen that the variation of ε” versus the applied frequency in the case of DOP, GPET, and polyester are so complicated and increase by incorporation these additives.

**Fig. 11:**
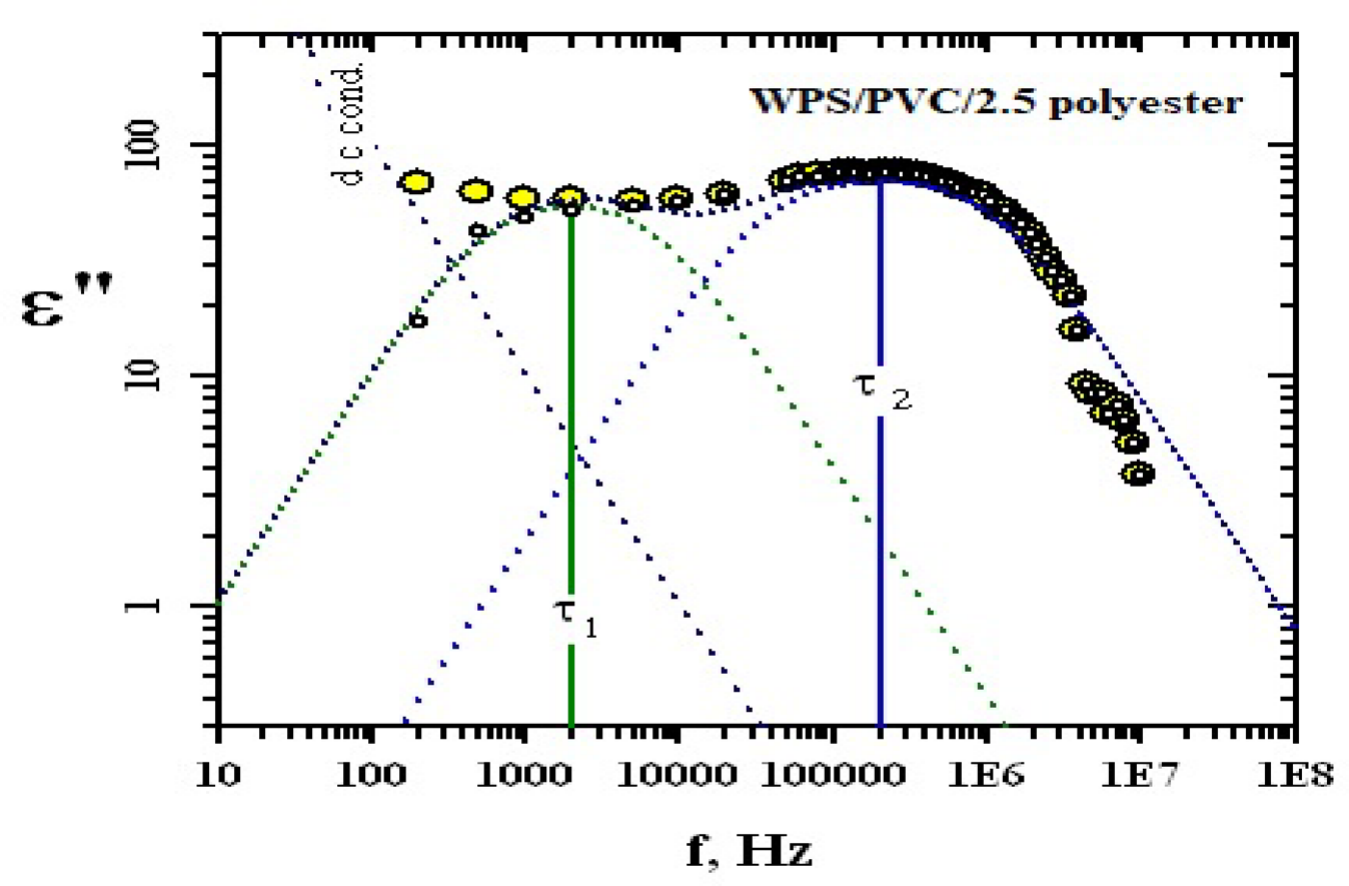
Example of the analyses for WPS/PVC/2.5polyester.

This complex behavior requires analysis based on the various well known dielectric functions^41–43^. After subtraction of the losses due to dc conductivity σ_dc_, The spectra were successfully fitted using two Fröhlich terms with distributions 3 and 4, respectively, an example of the analyses was given for WPS/PVC containing 2.5 polyester. As shown from Fig. 11, analysis of the WPS/PVC blend containing 2.5 phr polyester revealed two distinct relaxation processes, as evidenced by two separate peaks in the ε″ vs. frequency plot. These peaks correspond to relaxation times τ₁ and τ₂, associated with interfacial (Maxwell–Wagner–Sillars) and dipolar polarization, respectively. The first process τ_1_was found to be in the order of 10^− 5^ s and found to be fixed and does not affect by additives content. The second one τ_2_ which is our interest that reflects the interaction that may occur with the polymer matrix and type of plasticizer content was found to increase by decreasing either DOP or GPET content^34^. This decrease reflects the flexibility of the polymeric chain which makes it easy to be allied under the influence of the electric filed.

**Fig. 12:**
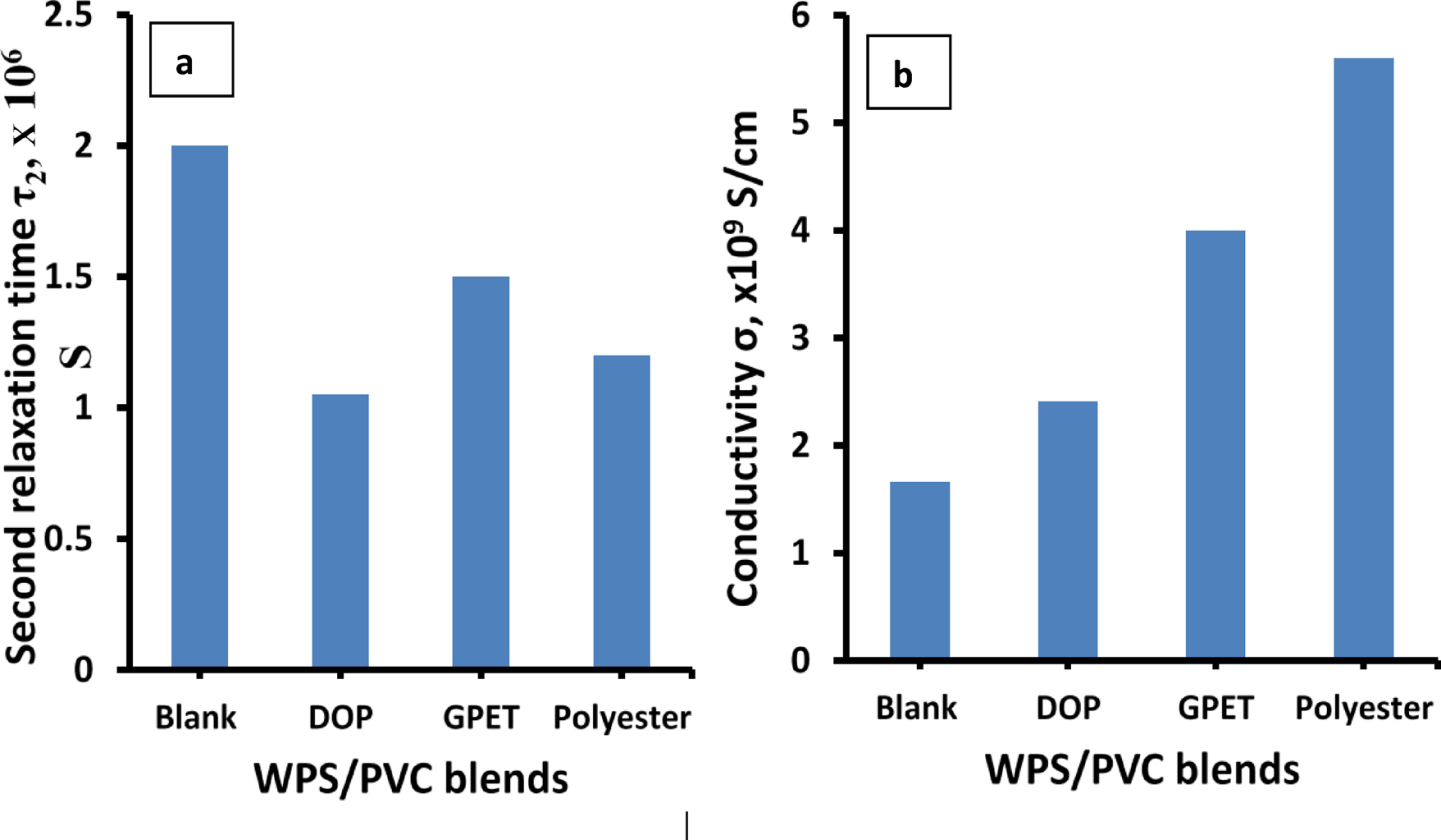
(**a**) Second relaxation timeτ_2 and_ (**b**) electrical conductivity σ for WPS/PVC blends.

The obtained values of τ_2_ are given in Fig. 12. Second relaxation time (τ_2_), decreases with the inclusion of additives when compared to the blank samples. A lower relaxation time suggests faster dipole response to the electric field. This behavior confirms that the additives, particularly polyester, reduce the energy barriers for dipole rotation and enhance the dielectric response. DOP and GPET also reduce τ, reflecting increased chain mobility and better phase interaction, but not as effectively as polyester.

The dc electrical conductivity σ_dc_ was calculated from the measured extrapolation of the measured resistance R using the relation.

$$\upsigma_{\mathrm{dc}} = \mathrm{l}/text{RA}$$

Where l is the sample thickness and A is its aria.

The obtained data are given in Fig. 12b.This figure indicates a clear increase in dc electrical conductivity (σ) with the addition of the three additives. Polyester again exhibits the greatest effect, followed by GPET and DOP. This is because the additives facilitate charge carrier movement by increasing polymer chain mobility and possibly introducing localized pathways for conduction. Polyester, with its polar groups and better compatibility, likely creates more continuous conductive domains, resulting in higher conductivity.

**Fig. 13:**
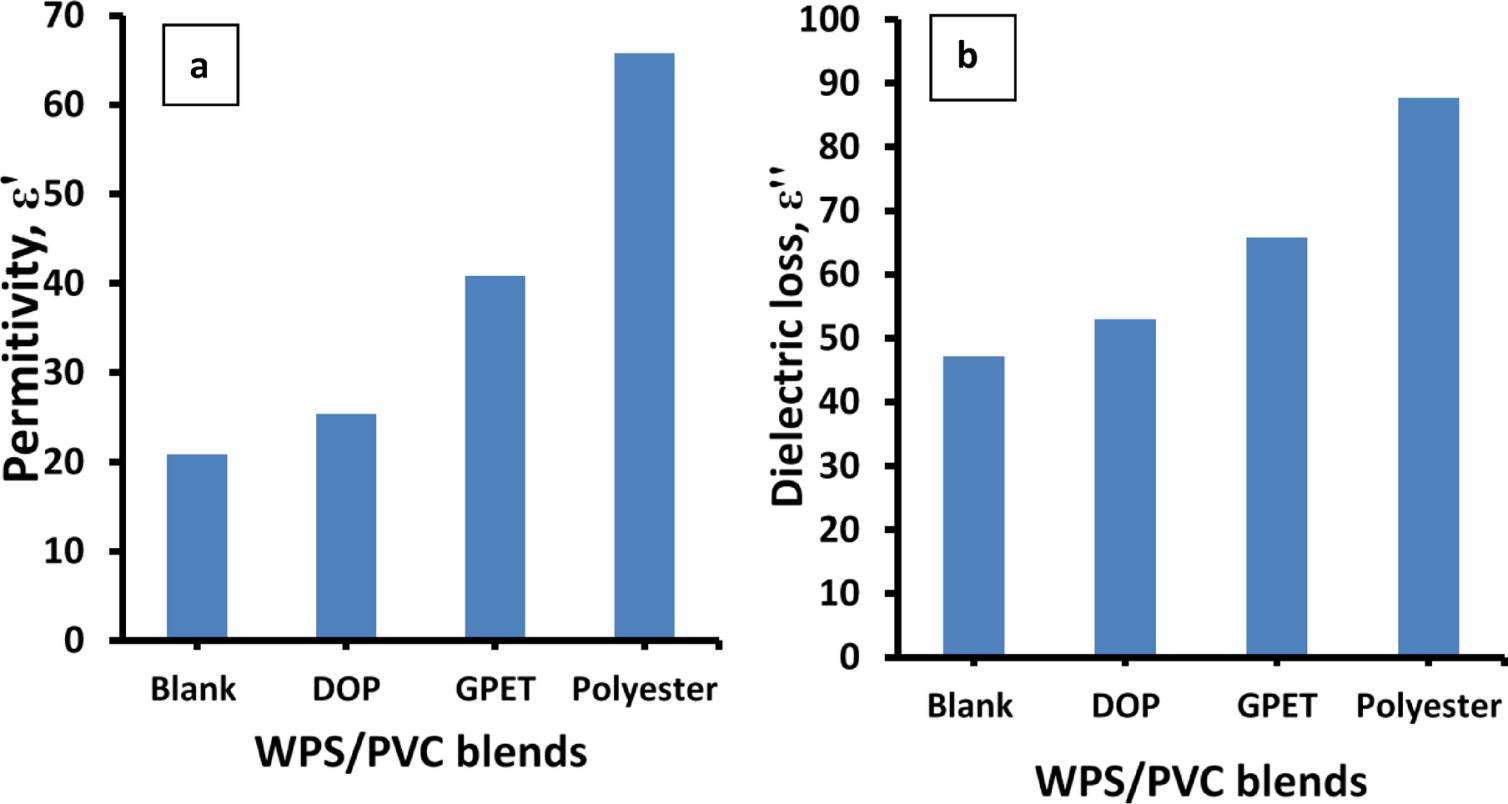
(**a**) Permittivity and (**b**) dielectric loss for WPS/PVC blends at fixed *f*, 100 Hz.

Figure 13(a-b) shows the change in permittivity (ε’) and dielectric loss (ε’’) for the various WPS/PVC blends at fixed frequency 100 Hz. From Fig. 13a, it is evident that all additives lead to an increase in ε’, with the polyester additive exhibiting the most significant enhancement. The increase in ε’ is attributed to improved polarization within the polymer matrix. The polyester, having polar functional groups and high chain mobility, enhances dipole alignment under the applied electric field. GPET and DOP also contribute to better dispersion and chain flexibility, promoting increased space charge polarization, though to a lesser extent than polyester. Also Fig. 13b shows that the dielectric loss increases with the addition of all additives, with polyester showing the highest value. This can be explained by the enhanced interfacial polarization and dipole reorientation losses due to the higher polarity and amorphous nature of the additives, especially polyester. GPET and DOP also raise the dielectric loss but not as significantly as polyester, indicating their moderate influence on energy dissipation mechanisms.

## Conclusion

In this study, the effects of various additives including DOP, GPET, and polyester synthesized from PET on the properties of WPS/PVC blends were thoroughly investigated. The results revealed that the addition of these modifiers significantly improved the melt flow rate, mechanical properties, and thermal behavior of the blends. The melt flow rate increased from 0.48 g/10 min (blank) to 0.52, 0.64, and 0.975 g/10 min for DOP, GPET, and polyester, respectively. Tensile strength also improved remarkably, reaching 21.96 MPa for GPET, compared to 10.6 MPa for the blank sample. Similarly, elongation at break rose from 12% to 37% with GPET, while hardness values slightly decreased due to the plasticizing effect of the additives. Thermal analysis showed that the burning rate dropped significantly from ~ 4 mm/s (blank) to ~ 0.5 mm/s with polyester, indicating improved flame retardancy. Additionally, the combustion energy decreased from 552 cal (blank) to around 404 cal with polyester, further confirming its flame-retardant properties.

Surface and cross-sectional morphological analysis demonstrated improved dispersion of silica and better phase compatibility, especially in samples containing GPET and polyester. Dielectric studies showed that all additives enhanced permittivity (ε′) and dielectric loss (ε″), with polyester showing the highest values due to strong interfacial polarization and dipolar activity. The second relaxation time (τ₂) decreased with the addition of additives, indicating greater dipole responsiveness and chain mobility. DC conductivity also increased, with polyester exhibiting the highest value, attributed to its polar structure and better matrix interaction. Overall, the incorporation of additives such as DOP, GPET, and polyester significantly enhanced the properties of WPS/PVC blends. Among them, polyester demonstrated the most pronounced improvement, offering higher tensile strength, elongation, and thermal stability, along with superior dielectric performance and flame retardancy. These improvements suggest that WPS/PVC containing polyester is particularly suitable for applications requiring both mechanical durability and safety, such as electrical insulation, construction materials, and automotive components. While DOP- and GPET-modified blends may be more appropriate for applications where moderate mechanical strength and thermal resistance are sufficient.

## Data Availability

All data generated or analyzed during this study are included in this published article and available from the corresponding author on reasonable request.
